# Single-Element Dual-Interferometer for Precision Inertial Sensing: Sub-Picometer Structural Stability and Performance as a Reference for Laser Frequency Stabilization

**DOI:** 10.3390/s23249758

**Published:** 2023-12-11

**Authors:** Victor Huarcaya, Miguel Dovale Álvarez, Kohei Yamamoto, Yichao Yang, Stefano Gozzo, Pablo Martínez Cano, Moritz Mehmet, Juan José Esteban Delgado, Jianjun Jia, Gerhard Heinzel

**Affiliations:** 1 Max-Planck-Institut für Gravitationsphysik (Albert-Einstein-Institut) and Institut für Gravitationsphysik, Leibniz Universität Hannover, Callinstrasse 38, D-30167 Hannover, Germany; 2 Laser Link (Shanghai) Aerospace Technology Co., Ltd., Shanghai 200433, China; 3 Department of Physics & Astronomy, Texas A&M University, College Station, TX 77843, USA; 4 Key Laboratory of Space Active Opto-Electronics Technology, Shanghai Institute of Technical Physics, Chinese Academy of Sciences, Shanghai 200083, China; 5 University of Chinese Academy of Sciences, Beijing 100049, China

**Keywords:** laser interferometry, inertial sensing, optical readout

## Abstract

Future GRACE-like geodesy missions could benefit from adopting accelerometer technology akin to that of the LISA Pathfinder, which employed laser interferometric readout at the sub-picometer level in addition to the conventional capacitive sensing, which is at best at the level of 100 pm. Improving accelerometer performance holds great potential to enhance the scientific output of forthcoming missions, carrying invaluable implications for research in climate, water resource management, and disaster risk reduction. To reach sub-picometer displacement sensing precision in the millihertz range, laser interferometers rely on suppression of laser-frequency noise by several orders of magnitude. Many optical frequency stabilization methods are available with varying levels of complexity, size, and performance. In this paper, we describe the performance of a Mach–Zehnder interferometer based on a compact monolithic optic. The setup consists of a commercial fiber injector, a custom-designed pentaprism used to split and recombine the laser beam, and two photoreceivers placed at the complementary output ports of the interferometer. The structural stability of the prism is transferred to the laser frequency via amplification, integration, and feedback of the balanced-detection signal, achieving a fractional frequency instability better than 6 parts in $10^{13}$, corresponding to an interferometer pathlength stability better than $1\;\mathrm{pm}/\;\sqrt{\mathrm{Hz}}$. The prism was designed to host a second interferometer to interrogate the position of a test mass. This optical scheme has been dubbed “single-element dual-interferometer” or SEDI.

## 1. Introduction

In the realm of high-precision inertial sensing, where tiny displacements can hold profound significance, the pursuit of ever-higher measurement sensitivity has driven innovation in optical metrology. Among these, laser interferometry has become a standard tool, particularly in the field of experimental gravitational physics, where it is used, e.g., to reveal the gravity field of Earth [1,2], understand climate change [3], or uncover new astrophysical systems through the observation of gravitational waves [4,5,6].

The GRACE Follow-On (Gravity Recovery and Climate Experiment Follow-On) mission, launched to orbit on 22 May 2018, measured changes in Earth’s gravitational field with unparalleled precision by deploying a pair of identical satellites in low Earth orbit [1]. These satellites used microwave and laser ranging instrumentation to detect variations in gravitational forces experienced as they orbited the planet [7]. Such variations are indicative of changes in mass distribution, offering insights into water storage, ice melt, and land movement. GRACE Follow-On has significantly contributed to understanding Earth’s water cycle, ice sheet dynamics, groundwater depletion, and other essential aspects of the Earth’s climate [3,8,9].

The accelerometers onboard each spacecraft are used to measure non-gravitational accelerations on the spacecraft in order to separate their effects from the effects of gravitational accelerations, which are the observables of interest. Non-gravitational forces include residual imbalanced thruster firings and non-gravitational environmental effects, such as atmospheric drag and solar and Earth radiation pressures.

Each accelerometer houses a test mass (TM), which is kept in a state of nearly free fall. The accelerometer monitors the position and motion of the TM using capacitive sensing by placing electrodes on both the TM and its housing. When the TM is perturbed due to inertial forces acting on it, the gap between the electrodes changes, altering the capacitance of the system. The accelerometer measures this change in capacitance, which can be related to the displacement of the TM with ∼$100\;\mathrm{pm}/\;\sqrt{\mathrm{Hz}}$ sensitivity.

The accelerometers are, at present, a dominant noise source in GRACE-like missions, with stray acceleration at the ∼$10^{-10}\;{\mathrm{ms}}^{-2}/\;\sqrt{\mathrm{Hz}}$ level [7,10,11]. They could be improved by using technology from the LISA Pathfinder mission [12], which demonstrated ∼$10^{-15}\;{\mathrm{ms}}^{-2}$ residual TM acceleration in interplanetary orbit [13,14], employing laser interferometric readout at the level of $1\;\mathrm{pm}/\;\sqrt{\mathrm{Hz}}$ displacement noise instead of electrostatic readout at ∼$100\;\mathrm{pm}/\;\sqrt{\mathrm{Hz}}$. A reduction in the TM acceleration noise can lead to an important improvement in the scientific return of future geodesy missions focusing on mass change, especially in a scenario with multiple pairs of geodesy satellites [2].

Laser interferometric inertial sensors aiming to measure TM displacements with sub-picometer precision over time scales of hundreds and even thousands of seconds rely on some form of reduction in laser-frequency noise, which couples through optical pathlength mismatches between the interfering arms. The most common methods of laser-frequency stabilization are locking to an ultra-stable optical cavity [15,16,17,18], or to an atomic or molecular transition [19,20]. These methods are costly, bulky, and rely on complex lock-acquisition schemes. An alternative technique is locking to a Mach–Zehnder interferometer (MZI) with several centimeters of optical pathlength difference [21]. By introducing an intentional arm length mismatch between the arms, the interferometer’s output signal acquires a sinusoidal dependence on the laser frequency, creating an opportunity for laser locking. Furthermore, the two output ports of the interferometer can be subtracted to derive a signal that conserves the sinusoidal dependence, but is largely insensitive to laser power fluctuations.

In a recent paper [22], laser-frequency stabilization via locking to an unequal-arm MZI was shown to provide a stability similar to that of high-performance reference lasers based on molecular iodine hyperfine transitions. By combining a quasi-monolithic interferometer and quasi-monolithic fiber injector with a very stable thermal environment, a fractional frequency instability on the level of a few parts in $10^{13}$ was achieved for measuring times up to 1000 s.

Despite the high performance for a system of that size, the MZI in [22] has one major drawback in that its manufacturing and assembly is comparatively complex. The interferometer consists of a custom quasi-monolithic fiber injector, five coated fused-silica components (two mirrors and three beam-splitters), and an ultra-stable glass ceramic baseplate. All of the optical components have to be bonded to the baseplate using UV adhesive in a time-consuming and delicate process. The component alignment is achieved using a coordinate measurement machine and a combination of template-assisted positioning for uncritical components and an adjustable pointing finger assembly for the critical recombination beam-splitter [23].

The custom fiber injector is itself complex, consisting of five components, four of which are custom-designed parts that also have to be bonded together using UV adhesive. The fiber injector has to be pre-assembled in another time-consuming and delicate process before it can be included in the full interferometer assembly.

In this paper, we present an unequal-arm Mach–Zehnder interferometer made of a single optical component that can reach sub-picometer displacement sensitivity at 2 mHz using a commercial fiber injector, on a package that is several times smaller than the interferometer presented in [22]. The construction of the prism can be outsourced to a company specializing in optics manufacturing, and its assembly in the laboratory is straightforward. This interferometer design was first presented in [24], and its performance is described here.

## 2. Experimental Setup

**Figure 1:**
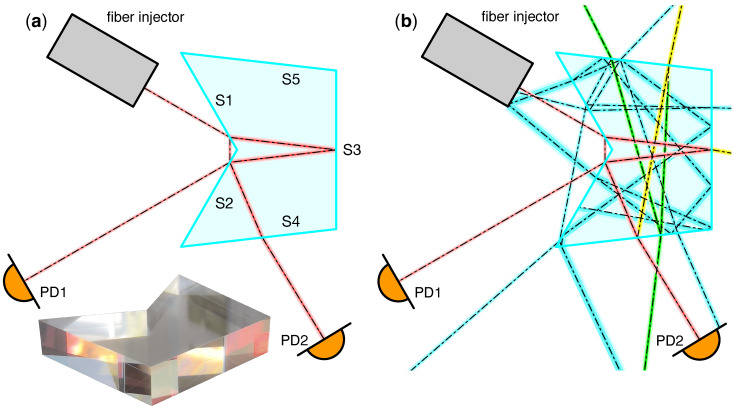
Single-element dual-interferometer (SEDI) prism showing only the reference interferometer. The prism consists of five optical surfaces with different coatings. Surfaces S1 and S2 split and recombine the beams. Surface S3 acts as a highly reflective mirror for the long arm beam, but is anti-reflective-coated on the sides. Surfaces S4 and S5 are anti-reflective-coated to minimize the impact of stray light in the setup. The main interferometric beams are shown in red (**a**). The prism geometry has been optimized via optical simulations to minimize the impact of ghost beams in the photodiodes. Ghost beams with $1\;>\;P_{\mathrm{ghost}}/P_{\mathrm{main}}\;>\;10^{-3}$ (yellow), $10^{-3}\;>\;P_{\mathrm{ghost}}/P_{\mathrm{main}}\;>\;10^{-7}$ (green), and $10^{-7}\;>\;P_{\mathrm{ghost}}/P_{\mathrm{main}}\;>\;10^{-12}$ (cyan) relative power levels are depicted in (**b**). Two detectors (PD1 and PD2) are placed at the complementary output ports of the interferometer to derive the balanced detection signal used for laser frequency stabilization.

The custom-designed pentaprism is shown in Figure 1. A monolithic piece of fused silica glass was formed through the optical contacting of two smaller prisms. A total of six coating runs were applied to the prism. Two 50:50 beam-splitter coatings were applied to surfaces S1 and S2. Surface S3 was first coated with an anti-reflective (AR) coating, except for a small portion of the surface in the middle, where it is coated with a highly reflective (HR) coating. Finally, two AR coatings were applied to surfaces S4 and S5.

The prism can form an unequal-arm Mach–Zehnder interferometer for a beam incident on S1 at a certain position and orientation. In the resulting interferometer, the short-arm beam propagates outside of the optic, while the long-arm beam propagates inside with an optical pathlength difference of $l_{\mathrm{ref}}=144$ mm between the two. The two beams are recombined at S2, and their interference is captured by the photodiodes PD1 and PD2, placed on the complementary output ports of the recombination beam-splitter. The power at the photodiodes depends on the laser frequency *f* and is given by

(1) $$\begin{array}{cc} P_{1}\left(f\right) & =p_{1}\left[1+c_{1}\cdot \cos\left(\frac{2\pi f\Delta l}{c}+\varphi_{0}\right)\right]\\ P_{2}\left(f\right) & =p_{2}\left[1-c_{2}\cdot \cos\left(\frac{2\pi f\Delta l}{c}+\varphi_{0}\right)\right] \end{array}$$

where $p_{1,2}$ are the optical powers at each photodetector in mid-fringe, $c_{1,2}$ are the interferometric contrasts at each photodetector, Δl is the interferometer’s optical path length difference, *c* is the speed of light, and $\varphi_{0}$ is an arbitrary constant. After a direct current subtraction, and trans-impedance amplification, the resulting signal is given by

(2) $$\begin{array}{cc} v(f) & =G\left[P_{1}\left(f\right)-P_{2}\left(f\right)\right]\\ \; & =G\left[p_{1}-p_{2}+\left(c_{1}p_{1}+c_{2}p_{2}\right)\cdot \cos\left(\frac{2\pi f\Delta l}{c}+\varphi_{0}\right)\right] \end{array}$$

where G[V/W] is the trans-impedance gain. If balanced operation is achieved, with nearly equal power levels on both photodiodes (i.e., $p_{1}=p_{2}$), the signal becomes

(3) $$v\left(f\right)=Gp_{1}\left(c_{1}+c_{2}\right)\cdot \cos\left(\frac{2\pi f\Delta l}{c}+\varphi_{0}\right)$$

Equation (3) has periodic zero crossings that are independent of the laser beam power and are used to lock the laser’s frequency. The slope of the error signal at the operating point is proportional to the available optical power, the interferometric contrasts, the trans-impedance gain, and the interferometer’s arm length difference.

A focusing lens is placed in front of each photodiode to minimize transverse beam walk, and thin-film polarizers with high extinction ratios are placed after the lens to mitigate the impact of stray light. Balanced operation is obtained by adjusting the polarizer’s rotation angles such that both photodiodes receive the same amount of laser power at the mid-fringe operating point.

In addition to the aforementioned interferometer, called the “reference interferometer” (Ref. IFO), the prism was designed to be injected with a second beam, derived from the same laser source, to form an additional interferometer called the “test mass interferometer” (TM IFO) [24]. The TM IFO phase would contain the test mass displacement signal which could be recovered by employing a suitable phase readout method [25,26]. Due to its characteristics, the prism was dubbed the “single-element dual-interferometer”, or SEDI.

**Figure 2:**
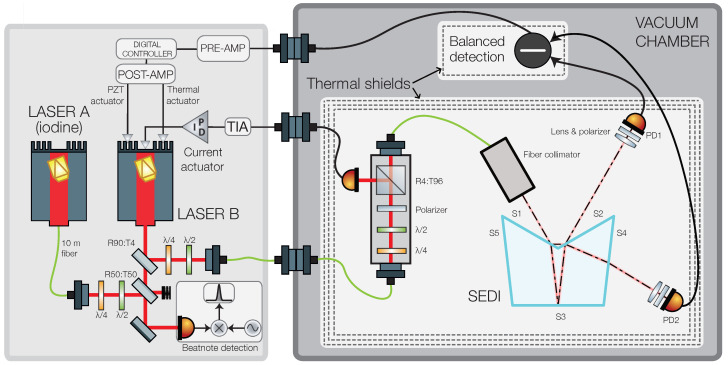
Experimental setup. The single-element dual-interferometer (SEDI) prism is surrounded by thermal shields and placed inside a vacuum chamber along with auxiliary optics for polarization adjustment and amplitude stabilization. A commercial fiber collimator is used to inject a beam derived from laser B into the SEDI reference interferometer. Two photodiodes are placed at the complementary output ports of the interferometer, with a focusing lens and thin-film polarizer placed in front that help mitigate known noise sources. The difference current between the two photodiodes is converted to a voltage via a low-noise, low-drift transimpedance amplifier. The amplifier signal is filtered, digitized, and used as input in a digital PI-controller to derive a control signal that is fed back to laser B’s fast and slow actuators, thereby transferring the interferometer’s pathlength stability to the laser frequency. A beatnote signal in the order of a few GHz is obtained by interfering laser B with a second, more stable laser (laser A). The beatnote signal is mixed-down to below 100 MHz using an ultra-stable GHz source, and read by a micro-cycle-stable phasemeter to characterize the achieved stability.

To characterize the performance of the reference interferometer in the SEDI, we use the experimental setup depicted in Figure 2. Light from a 1064 nm non-planar ring oscillator laser (laser B) is split two ways, with one part being fed to a vacuum chamber containing the SEDI prism, and the remaining part being interfered with a reference 1064 nm laser (laser A) that is locked to a molecular iodine hyperfine transition (R(56)32-0 ‘a1’).

Inside the vacuum chamber, the light is first injected into a small bench where a combination of retarder waveplates and a polarizer produce s-polarized light. A small portion of the light is captured by an auxiliary photodiode and used for stabilization of the laser amplitude, and the rest is coupled back into a fiber and injected into the SEDI prism’s Ref. IFO via a commercial fiber coupler. All of the aforementioned components are mounted on an aluminum breadboard and surrounded by a high-performance multi-layer thermal shield similar to the one described in [27].

The photodiodes are operated in reverse bias voltage and connected in a balanced differential trans-impedance amplifier (TIA) performing a direct current subtraction, giving rise to a signal with sinusoidal dependence on the laser frequency and the interferometer’s pathlength noise. The TIA is surrounded by a separate thermal shield to avoid temperature cross-couplings between the optics and electronics.

The TIA output signal is low-pass-filtered and enhanced by a pre-amplifier before being digitized by a Moku:Lab instrument [28] and used as an error signal in a digital PI-controller. The resulting control signal is filtered by a post-amplifier and fed back to the laser via both a slow thermal actuator and a high-speed piezo-electric transducer actuator.

The beatnote between lasers A and B is in the GHz regime, and thus it is down-mixed to below 100 MHz by an ultra-stable GHz signal generator (SMB100A instrument by Rohde & Schwarz [29]) before being read out by a Moku:Lab instrument acting as a phasemeter.

## 3. Results

**Figure 3:**
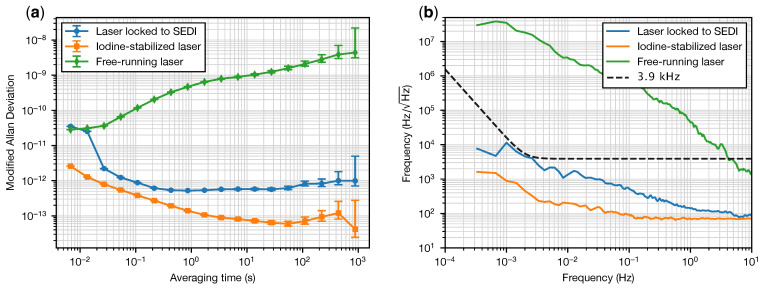
Modified Allan deviations (**a**) and frequency spectral densities (**b**) of laser B when it is free-running (green), laser B when it is locked to the SEDI reference interferometer (blue), and laser A, which is used as reference (orange). The picometer-equivalent frequency noise for a 14.4 cm interferometer is represented by a black dashed curve at 3.9 kHz$/\;\sqrt{\mathrm{Hz}}\times u\left(f\right)$. The error bars in (**a**) are computed using the “finite differences” method [32].

The frequency spectral density [30] and modified Allan deviation [31] of the beatnote between the laser locked to the SEDI and the iodine-stabilized reference laser are shown in Figure 3a and Figure 3b, respectively, for a typical 1 h measurement at a rate of 150 samples per second (blue curves). Also shown are the free-running noise of the laser (green) and the noise of the reference laser (orange). Figure 3b also shows the frequency noise spectral density of 3.9 kHz$/\;\sqrt{\mathrm{Hz}}\times u\left(f\right)$ (black dashed line), representing the picometer-equivalent frequency instability of an interferometer with 14.4 cm arm length difference, as is the case in the SEDI Ref. IFO. The noise envelope function

(4) $$u\left(f\right)=\sqrt{1+{\left(\frac{2\;\mathrm{mHz}}{f}\right)}^{4}}$$

is used frequently to scale the sensitivity requirements for inertial sensing of freely floating test masses in space. It describes a mixture of white noise with a flat power spectrum at frequencies larger than 2 mHz, and random run noise with $f^{-4}$ power spectrum at lower frequencies, where it is expected that the acceleration noise of the test mass becomes dominant.

The modified Allan deviation is chosen for its ability to distinguish between white and flicker phase noise at short averaging times (i.e., at short $\tau =m\tau_{0}$, where τ is the averaging time, $\tau_{0}$ is the gate time or sampling time, and *m* is the averaging factor), or equivalently at high frequencies. This function is also widely used in the frequency standards community, such that our results may be easily compared with other references.

Inspection of Figure 3a,b, which provide largely the same information, reveals that the noise of the reference laser (laser A) is low enough compared to the laser under test (laser B) and that its instability can be neglected in the estimation of the noise of the unit under test. The laser locked to the SEDI prism presents a fractional frequency instability below the $10^{-12}$ level for averaging times between 0.1 and 1000 s. This performance is similar to what can be expected from high-performance iodine-stabilized reference systems.

**Figure 4:**
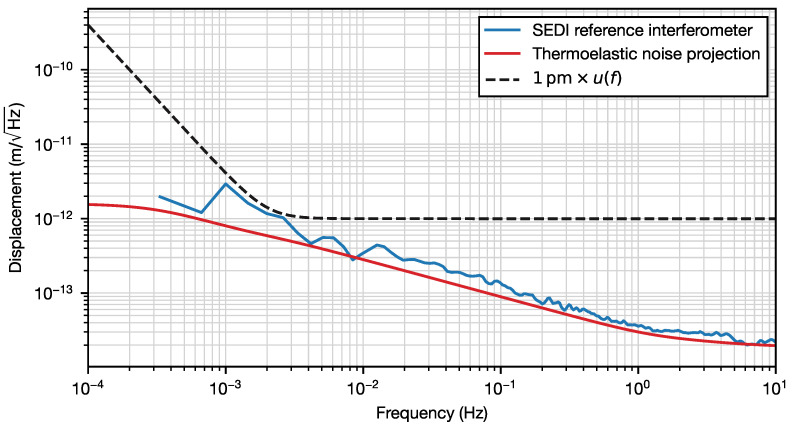
Amplitude spectral density of the SEDI reference interferometer pathlength (blue) and projection of the thermoelastic noise (red). A displacement noise of 1 pm$/\;\sqrt{\mathrm{Hz}}\times u\left(f\right)$ is represented by the black dashed curve.

We convert the measured fractional frequency instability into equivalent pathlength noise by invoking

(5) $$\frac{\Delta (l_{\mathrm{ref}})}{l_{\mathrm{ref}}}=\frac{\Delta f}{f_{0}},$$

where $f_{0}$ is the average laser frequency (roughly 282 THz). The resulting pathlength noise is shown in Figure 4, together with a projection of thermoelastic noise obtained via numerical modeling, using the model described in [24] and assuming a uniform temperature distribution of $20\;\mu K/\sqrt{\mathrm{Hz}}$ spectral density in the surface of the prism, which is consistent with our measurements.

The test mass displacement sensitivity of the SEDI test mass interferometer can be estimated by multiplying the measured reference interferometer pathlength noise by the ratio between armlength differences in both interferometers (i.e., approximately 3.5). This results in $10\;\mathrm{pm}/\sqrt{\mathrm{Hz}}$ sensitivity at 1 mHz, $1\;\mathrm{pm}/\sqrt{\mathrm{Hz}}$ sensitivity at 10 mHz, and $0.1\;\mathrm{pm}/\sqrt{\mathrm{Hz}}$ sensitivity at 1 Hz, which is in good agreement with the noise budget analysis presented in [24].

A comparison of the projected SEDI test mass displacement noise to previous works on compact interferometric inertial sensors is given in Table 1. For additional references, including earlier works, see the comprehensive overview by Watchi et al. [33] on compact interferometers.

## 4. Conclusions

A Mach–Zehnder interferometer with unequal arm lengths was implemented in a custom pentaprism formed by single piece of fused silica glass. The optic was constructed by the optical contacting of two smaller prisms and applying six coating runs to the five optical surfaces. The interferometer’s balanced detection signal was used to lock the frequency of a laser down to a fractional instability below $10^{-12}$ for averaging times between 0.1 and 1000 s. The equivalent pathlength stability of the interferometer is better than 1 pm$/\;\sqrt{\mathrm{Hz}}\times u\left(f\right)$ from 1 mHz to 10 Hz.

This paper presents the first experimental demonstration of the SEDI optic, whose design was introduced in [24]. The performance characterization of the reference interferometer in the SEDI is vital before attempting the next step: the dual-interferometer configuration. From the noise model described in [24], and assuming that the Ref. IFO and TM IFO phase noises are uncorrelated, we expect the upper bound of the Ref. IFO contribution to the test mass displacement noise to be 10 pm at 1 mHz and 1 pm at 10 mHz. This contribution is dominated by thermoelastic deformation. The actual noise contribution may be lower, as we expect there to be some level of coherence in the thermal noise of the two interferometers.

The advantage of the single-element interferometer over the multi-element approach is the ease of manufacture and assembly. While the Mach–Zehnder interferometer presented in [22] requires specialized assembly procedures, the SEDI prism does not. We expect that the difference in their performance comes down to thermoelastic noise, which could be lower in [22] due to the use of the ultra-low-expansion glass ceramic baseplate.

Unequal-arm Mach–Zehnder interferometers are an attractive solution for laser-frequency stabilization in a compact setup. In contrast to cavity-locking and atomic or molecular references, the technique offers a wide operating range and does not rely on complex lock acquisition procedures. Since the Mach–Zehnder can be integrated as part of the optical bench in future gravity missions already featuring an optical bench assembly, it holds the potential to eliminate the need for a separate laser stabilization subsystem.

## Figures and Tables

**Table 1:** Comparison of the sensitivity of compact interferometers in recent works. ${}^{†}$ In this work, a separate frequency reference interferometer is used to reduce the laser frequency noise. ${}^{††}$ The noise is projected from the measured reference interferometer noise floor.

		Noise at 1 mHz	Noise at 1 Hz	Wavelength	Dimensions
Year	Device	(pm/$\sqrt{\mathrm{Hz}}$)	$\left(\mathrm{pm}/\sqrt{\mathrm{Hz}}\right)$	(nm)	(cm)
2018	Pisani [34]	$2\times 10^{3}$	0.6	1064	11.0×10.0×6.0
2019	Isleif [35] ${}^{†}$	20	0.23	1064	2.5×2.5×2.5
2022	Yan [36]	$10^{4}$	4–6	1064	20.0×20.0×7.0
2022	Zhang [37]	$10^{3}$	0.6	1550	2.0×2.0×1.0
2022	Smetana [38]	-	0.3	1064	1.3×0.4
2022	Kranzhoff [39]	$10^{9}$	1	1064	32.0×23.0×31.0
2023	Huarcaya [22]	0.4	0.007	1064	13.5×13.5×7.1
2023	SEDI (this work) ${}^{††}$	10.2	0.12	1064	9.8×7.8×2.0

## Data Availability

Data are contained within the paper.
